# Retraction: Catestatin Increases the Expression of Anti-Apoptotic and Pro-Angiogenetic Factors in the Post-Ischemic Hypertrophied Heart of SHR

**DOI:** 10.1371/journal.pone.0246900

**Published:** 2021-02-04

**Authors:** The PLOS ONE Editors

Following the publication of this article [1], concerns were raised regarding the results presented in Figure 6. Specifically, the following results appear similar:

- Lane 2 of the p-ERK1/2 panel, Lane 1 of the ERK1/2 panel, lane 2 of the ERK1/2 panel, and lane 3 of the ERK1/2 panel when flipped.
- Lane 1 and 2 of the p-PKCε panel and lane 1 and 2 of the PKCε panel when flipped.
- The β-Actin panels for the PKCε and GSK-3β samples
- Lane 3 of the p-PKCε and Lanes 3 of the β-Actin panels (PKCε and GSK-3β) when flipped.
- Lane 1 and lane 3 of the GSK-3β panel.

The corresponding author commented that the wrong image was inadvertently included in the published article as Figure 6. The authors offered to replace Figure 6 with a corrected version and provided replicate image data to support the Figure 6 results. They also noted that the experimental and control data for Figure 6 were obtained on parallel blots. However, the information and data provided did not clarify the above concerns, and as such concerns remain about the integrity and reliability of the published figure.

In light of the unresolved concerns about Figure 6, the *PLOS ONE* Editors retract this article.

CP, TP, DA, MGP, CA, SKM, BT, PP, MCC, and TA agreed with the retraction, stand by the article’s findings, and apologise for the issues with the published article. FT either did not respond directly or could not be reached.

In addition, during the assessment of these concerns it came to light that there was a potential competing interest between the handling Academic Editor and one or more authors. We regret that this was not identified and addressed prior to the article’s publication.
